# Traditional and religious treatments administered to children with autism spectrum disorder in South Africa: a qualitative study of practices, composition and risk

**DOI:** 10.3389/fneur.2026.1946904

**Published:** 2026-10-30

**Authors:** Ayanda Simelane, Curwyn Mapaling

**Affiliations:** 1 Community Psychosocial Research (COMPRES), North-West University, Potchefstroom, South Africa; 2 Optentia Research Unit, North-West University, Potchefstroom, South Africa

**Keywords:** autism spectrum disorder, faith healing, health regulation, qualitative research, South Africa, traditional medicine, treatment safety

## Abstract

**Introduction:**

The appraisal of treatments for autism spectrum disorder (ASD) has largely focused on interventions delivered within formal healthcare systems, particularly in high-income settings. In South Africa, children with ASD may receive concurrent care from hospitals, traditional health practitioners and religious leaders, yet the practices administered outside formal healthcare remain poorly documented. This study aimed to identify these practices and interpret their potential risks in relation to published evidence of harm.

**Methods:**

A qualitative study was conducted at a public tertiary hospital in Soweto, Johannesburg. Semi-structured interviews were undertaken with 24 parents and caregivers and 10 hospital clinicians, alongside two focus groups involving traditional health practitioners and religious leaders, respectively. As practitioner participants did not recognise the term autism, they were briefed on its DSM-5 features before discussing practices previously administered to children presenting with similar characteristics. Their accounts therefore did not necessarily concern children with confirmed ASD diagnoses. Data were analysed using reflexive thematic analysis, followed by deductive recoding of reported practices into a route-of-administration taxonomy.

**Results:**

Twenty-five distinct practices were identified. All children in the sample had received at least one intervention outside formal healthcare, with most receiving several alongside hospital treatment. Ingested and rectally administered preparations presented particular safety concerns because their constituents were undisclosed, dosing relied on clinical impression and paediatric formulations were unavailable. Neither caregivers nor clinicians could identify the substances administered, while practitioners declined to disclose their composition. Practitioners categorised ASD-related presentations within a broader conception of insanity, and one non-clinician proposed cutting tissue beneath a child's tongue to facilitate speech. Reported benefits, including explanatory frameworks, family connectedness and sustained hope, related primarily to caregivers' experiences rather than documented therapeutic improvements in children.

**Discussion:**

The findings identify potentially harmful and insufficiently documented interventions administered alongside formal ASD care. Their safety cannot be adequately assessed without information about their constituents and administration. Route-based risk triage, non-punitive disclosure during clinical consultations and protected constituent disclosure are recommended. As the study was conducted at a single urban tertiary hospital, its findings are context-specific and should not be generalised beyond the setting investigated.

## 1 Introduction

An estimated 61.8 million people were on the autism spectrum globally in 2021, or one in every 127, and autism spectrum disorder (ASD) ranks among the ten leading causes of non-fatal health burden in people under twenty years of age (1). Treatment has developed alongside a refined definition of the condition, better identification of comorbidities and improved measurement, and the evidence supporting it remains uneven. Two agents carry regulatory approval in the United States, both indicated for irritability, which is a comorbid presentation rather than a core feature, so pharmacotherapy addresses the difficulties that accompany ASD more than the difficulties that define it. Behavioral and developmental interventions carry the strongest support for core features, and the most recent synthesis of that literature reports modest effects that attenuate when caregiver-report outcomes are removed, together with the observation that benefits cannot be weighed against harms because harms are inadequately monitored across the field (2).

That appraisal literature shares a boundary condition worth making explicit. It describes interventions delivered inside formal health systems, and it is generated overwhelmingly in high-income settings. The majority of children with ASD live outside those settings. In much of sub-Saharan Africa a child with ASD may move between a public health system with limited developmental capacity and an extensive parallel sector of traditional and religious healing (3, 4), and the treatments delivered in that second sector fall outside the reach of any appraisal exercise conducted so far.

South Africa illustrates the position sharply. Pediatric developmental services concentrate in a small number of urban tertiary centers, diagnostic pathways are long, and specialist capacity is scarce, so families in the public sector wait. Alongside this sits a traditional health sector estimated at roughly 300,000 practitioners nationally, together with a large and heterogeneous religious healing sector in which claims to cure illness are made openly and are not subject to professional oversight (5). Consultation across these systems is ordinary rather than exceptional, and South African practitioners have themselves identified a need for biomedical knowledge and skills (6).

Existing research on ASD in African settings has established that families follow pluralistic pathways to care and that supernatural attributions of cause are common (3). On the Kenyan coast, parents and professionals attributed ASD to preternatural as well as biological causes and described treatment pathways running from traditional and spiritual healing to formal health facilities (7). Among Ethiopian caregivers of children with developmental disorders, supernatural explanatory models were widespread and traditional or religious healing had been sought alongside hospital care (8). In Nigeria, a substantial share of health workers themselves endorsed supernatural etiologies for childhood autism (9). In South Africa, the use of traditional remedies in children is well documented in general pediatric populations (10), although not in children with ASD. What that work has documented is help-seeking behavior and belief. What it has not documented is treatment. We are not aware of any study that establishes which substances children with a confirmed diagnosis of ASD receive from traditional and religious practitioners, by what route those substances enter the body, at what dose, or on whose authority. Without that information a whole category of pediatric exposure sits outside the evidence base, and it does so at the point where exposure is most likely to cause harm, since the risk of a preparation follows from its composition and its route rather than from the belief system that recommends it.

The harm literature is similarly uneven. For unconventional treatment of ASD specifically, the available evidence on adverse effects comes almost entirely from complementary and alternative medicine in high-income settings, where use is common and safety reporting is poor (11). We are aware of a single published case of a child with ASD harmed by a traditional preparation, a hyponatraemic seizure following herbal medicine given with large volumes of water by enema (12). For traditional treatments given to children in Southern Africa more generally, there is an older but consistent clinical literature. Traditional medicines were the second commonest cause of acute poisoning admissions in one Southern African series and the largest single contributor to poisoning deaths (13). A rural case series described a pediatric enema syndrome with in-hospital mortality above a quarter (14), and pediatric surgeons in Johannesburg continue to report children requiring major surgery for colonic injury after traditional enemas (15). None of this literature concerns children with ASD, and none of it identifies what was administered. The safety of traditional and complementary medicines in Africa has been described as largely unexamined (16), and for the population studied here that remains the position.

The policy context has moved faster than the evidence. The Global Traditional Medicine Strategy 2025 to 2034, adopted by the Seventy-eighth World Health Assembly, sets four objectives that include strengthening the evidence base, ensuring safety and regulation, and integrating traditional, complementary and integrative medicine into health systems, and it identifies the misappropriation of traditional knowledge as an obstacle to that work (17). South Africa has stated a commitment to integration consistent with that strategy (18). Its enabling legislation, the Traditional Health Practitioners Act 22 of 2007, was passed to provide a regulatory framework for the safety, quality and efficacy of traditional health care (19). Nineteen years on, the regulations required to bring the Act into operation remain unpromulgated, and a parliamentary committee found in May 2026 that no functional national register of practitioners exists and that enforcement mechanisms are absent (5). Integration is therefore national and international policy, and the evidence on which safe integration would depend has not been assembled.

This article reports the empirical component of a study conducted at a public tertiary hospital in Soweto, Johannesburg. Rather than ask what families believe, we asked what children receive. We interviewed parents and caregivers of children with a confirmed diagnosis of ASD, interviewed the hospital clinicians treating those children, and held focus groups with traditional health practitioners and with religious leaders, so that each practice could be described from more than one position. We then classified every practice reported by any participant according to how it enters the child's body.

Our objectives were threefold. The first was to document the traditional and religious practices administered to children with a confirmed diagnosis of ASD in this setting, and to classify them by route of administration. The second was to establish what is known about the composition, dosing and provenance of the preparations involved, and to identify where that knowledge is held and where it is absent. The third was to interpret the resulting exposure in the light of the published harm literature, as a contextual reading rather than a systematic appraisal, and to consider what would be required in this setting for the safe and regulated integration that current policy anticipates.

## 2 Materials and methods

### 2.1 Design

This article reports the empirical component of a larger doctoral study conducted at a public tertiary hospital in Soweto, Johannesburg (20). The doctoral study also comprised a normative component, in which the ethical responsibilities arising from the empirical findings were analyzed philosophically. That component is not reported here, and its bearing on which interview material appears in this article is set out in Section 2.5.3. The design was qualitative, exploratory and descriptive. We chose it because the treatments at issue have no documented evidence base, no standardized nomenclature and no existing clinical taxonomy in the South African literature, which rules out the designs ordinarily used to appraise treatment efficacy. Where a treatment landscape has not yet been described, description is the prior step.

Our purpose was to establish what children with a confirmed diagnosis of ASD are given outside the formal health system, by whom, on what reasoning, and with what consequences as reported by those closest to the administration of these treatments. We sampled four groups so that each practice could be described from more than one vantage point: the parents and caregivers who seek and consent to the treatments, the hospital clinicians who see the children before and afterwards, and the two categories of practitioner who provide the treatments.

Reporting follows the Consolidated Criteria for Reporting Qualitative Research (COREQ) (21). The completed 32-item checklist is provided as Supplementary File 1.

### 2.2 Setting

Data were collected at Chris Hani Baragwanath Academic Hospital (CHBAH), a public tertiary and quaternary hospital serving Soweto and referral cases from across Gauteng and from neighboring countries. The hospital is a teaching hospital of the University of the Witwatersrand and is among the largest in the world by bed count. Its catchment is predominantly Black African and predominantly reliant on the public sector, since private pediatric developmental services in Johannesburg are largely unaffordable to this population.

Participants were recruited from three services that see children with suspected or confirmed ASD: the psychology unit, the speech and occupational therapy unit, and the neurodevelopmental unit. At the time of data collection one psychiatrist served the psychology unit, and the pathway from first presentation to a confirmed diagnosis of ASD took up to six months where the child had already reached CHBAH, and considerably longer where the child had first presented at a district clinic. This diagnostic interval is relevant to the findings and we return to it in the discussion.

### 2.3 Participants and sampling

Non-probability purposive sampling was used to identify information-rich participants, followed by snowball referral among parents. Twenty-four parents and caregivers of children with a confirmed diagnosis of ASD took part in individual semi-structured interviews, comprising 22 biological parents and two other primary caregivers. Ten hospital clinicians took part in individual semi-structured interviews: one psychiatrist, three psychologists, three occupational therapists and three speech and language therapists. Two focus groups were held, one with twelve traditional health practitioners and one with twelve religious leaders.

Sample size was not fixed in advance, and we judge its adequacy retrospectively using the concept of information power (22). The aim of the study was narrow, to document practices administered to a defined clinical population at a single site. Sample specificity was high, since every parent had a child with a hospital-confirmed diagnosis and every clinician had treated such children. Two considerations pointed the other way. The study drew on no established theory of these practices, and dialogue quality was uneven, since some practitioners declined to answer questions on composition. The analysis sought description across cases rather than depth within a few. Weighed together, these considerations support the judgement that 24 parent interviews, ten clinician interviews and two extended focus groups held sufficient information power for the descriptive aim of the study. Refusals among parents and clinicians were not systematically recorded, and we note this as a limitation. The instances of non-participation that the first author's records do show are that one father who had agreed to a joint interview with his partner withdrew shortly after it began and left her to continue alone, and that the interfaith body reported that most of the Hindu and Muslim representatives it invited did not respond.

Table 1 sets out the characteristics of the four cohorts. The demographic form completed before each parent interview recorded the child's pseudonym, sex, population group and date of diagnosis, and whether the child had been through traditional or religious rituals in relation to the condition (see Supplementary File 2). It did not record the age, education, employment or home language of the participant, and we therefore cannot report those characteristics. All parent and caregiver participants were Black African. Most were mothers and most described themselves as single parents; two were grandmothers acting as primary caregivers, and one couple was interviewed together. Of the children whose sex was recorded, 17 were boys and five were girls. The ten clinicians were drawn from the three participating units, and the psychiatrist was the only participant whose scope of practice permitted a formal diagnosis. The traditional health practitioner group comprised *izangoma, izinyanga* and *abathandazi* in proportions that were not recorded, and most of the active contributors to that discussion were men. The religious leaders were pastors, prophets and reverends drawn from an interfaith body in Gauteng; all but one were from Christian denominations, and one was Muslim.

**Table 1:** Characteristics of participants, by cohort.

Cohort	*n*	Data collection	Characteristics recorded
Parents and caregivers	24	Individual semi-structured interviews, in English with switching into isiZulu or Sesotho	22 biological parents and two other primary caregivers (grandmothers); predominantly mothers; most single parents; one couple interviewed jointly; all Black African. Children: 17 boys and five girls where sex was recorded; all with a hospital-confirmed diagnosis of ASD; most described as having limited or absent expressive speech. Participant age, education and employment not recorded.
Hospital clinicians	10	Individual semi-structured interviews, in English	One psychiatrist, three psychologists, three occupational therapists and three speech and language therapists; recruited from the psychology, speech and occupational therapy, and neurodevelopmental units; all had treated at least one child with a confirmed diagnosis of ASD. Age, sex and years of practice not recorded.
Traditional health practitioners	12	One focus group, two to three hours, in English and isiZulu	Recruited through one Johannesburg practitioner organization; included *izangoma* (diviners), *izinyanga* (herbalists) and *abathandazi* (faith healers), numbers in each category not recorded; most active contributors were men; the group leader was addressed as Doctor and senior men as Baba. Briefed on DSM-5 features of ASD before discussion.
Religious leaders	12	One focus group, two to three hours, in English	Recruited through one interfaith body in Gauteng; pastors, prophets and reverends; all but one from Christian denominations, one Muslim; invited Hindu and other representatives did not respond. Briefed on DSM-5 features of ASD before discussion.

Practitioner cohorts described children they recognized from the DSM-5 description provided before the discussion, rather than children with an independently confirmed diagnosis (see Sections 2.3 and 4.4).

Severity level under the Diagnostic and Statistical Manual of Mental Disorders, fifth edition [DSM-5; American Psychiatric Association (APA), (23)], was not systematically recorded on the study documentation, and we do not report it. Where the presentation of individual children is described in the findings, it rests on the accounts of parents and treating clinicians rather than on a formal severity rating. Most parents described children with limited or absent expressive speech at the time of interview, and this was the feature most often given as the reason for first seeking help.

Children were eligible where a diagnosis of ASD had been confirmed by the hospital. Diagnosis was verified against the hospital record and against a demographic form completed before the interview, and parents were screened twice, first by the referring clinician and again by the researcher on arrival. Clinicians were eligible where they had previously treated at least one child with a confirmed diagnosis of ASD. Traditional health practitioners and religious leaders were eligible where they had treated, or could describe treating, a child presenting with the features of ASD.

Practitioner recruitment proceeded through two Johannesburg-based organizations, one representing traditional health practitioners and one an interfaith body. Identifying organizations with functioning structures rather than registration alone proved difficult, and this difficulty is itself a finding that bears on the regulatory argument developed later. The traditional healer group included *izangoma* (diviners, who identify the cause of illness through communication with ancestors), *izinyanga* (herbalists, who prepare and dispense plant-based medicines) and *abathandazi* (faith healers, who work through prayer, often within African-initiated churches). *Abathandazi* work across both traditions, and their inclusion in this group follows the recruiting organisation's own categorization rather than a judgement of ours. The religious leaders group was drawn predominantly from Christian denominations and included one Muslim participant. The interfaith body reported that most invited Hindu and Muslim representatives did not respond.

One design feature requires emphasis because it constrains how the practitioner data may be read. Neither practitioner group recognized the term autism at the outset. Both organizations requested, and were given, a structured explanation of ASD drawn from DSM-5 criteria before discussion began. Practitioner accounts therefore concern children whom participants recognized from a clinical description, and not children with independently confirmed diagnoses. We treat their accounts as evidence of what is administered to children presenting with these features, and not as evidence of prevalence or of diagnostic concordance. The procedure also introduces a specific form of response bias, which we set out here rather than only in the limitations. Offering an external, biomedical description of a condition before asking practitioners to recall cases may shape which children they bring to mind and how they describe them, and it invites participants to fit remembered presentations to the description offered. The cases practitioners described therefore cannot be assumed to correspond to presentations they would have identified or grouped together unprompted, and the frequency with which they reported seeing such children is not interpretable. Two features of the procedure limit the effect without removing it. The briefing was confined to the diagnostic features of ASD and did not refer to any form of treatment, so the practices practitioners went on to describe were not suggested to them. The first item in the focus group guide asked whether participants had treated such a child, before any item asked what was done, so that the recall of cases preceded the discussion of practice. We did not test concordance between the briefing and practitioners' own categories beyond the finding, reported in Section 3.8, that they located the presentation within the broader category of *uhlanya*. We return to this in Section 4.4.

### 2.4 Data collection

Three semi-structured interview guides were developed from the literature, one each for parents, clinicians and the focus groups. All three were reviewed by the first author's doctoral supervisor and approved by the ethics committee before use, and they are reproduced in full in Supplementary File 3. Table 2 summarizes the domains each guide covered, the number of items in each domain, and whether the material generated is reported in this article or was reserved for the normative component of the doctoral study.

**Table 2:** Domains covered by the three interview guides and where the resulting material is reported.

Guide	Domain	Items	Reported
Parents (23 items)	Developmental history and diagnostic pathway	1	This article (Sections 3.1, 3.8)
	Cultural practices held by the family, practices attempted, perceived benefit and harm, reasons for not attempting	2 to 7	This article (Sections 3.1 to 3.9)
	Religious practices held by the family, practices attempted, perceived benefit and harm, reasons for not attempting	8 to 13	This article (Sections 3.1 to 3.9)
	What society and institutions should do to protect children from harmful practices; criteria for judging a practice right or wrong; how to improve beneficial practices	14 to 23	Normative component of the thesis; drawn on in Section 4.2.6 only as participant opinion
Clinicians (14 items)	Involvement with children with ASD and the diagnostic process	1 to 3	This article (Sections 2.2, 3.8)
	Parental response to diagnosis and rejection of diagnosis	4 to 6	This article (Section 3.8)
	Alternative treatments sought by families, reasons, and what is disclosed	7 to 10	This article (Sections 3.2 to 3.8)
	Whether seeking alternatives is acceptable; implications for wellbeing; who is obligated to protect children	11 to 14	Normative component of the thesis; drawn on in Sections 4.2.3 and 4.2.6 only as participant opinion
Focus groups (13 items, preceded by briefing)	Briefing on DSM-5 features of ASD; recognition of cases treated; frequency of consultation; who brings the child	Briefing, 1 to 3	This article (Sections 2.3, 3.8)
	Attributed causes and what parents are told	4 to 5	This article (Sections 3.7, 3.8)
	Treatments advised	6	This article (Sections 3.1 to 3.7)
	Perceived benefit and harm of rituals and how harm is prevented	7 to 11	This article (Sections 3.3, 3.8) for descriptions of practice; normative material reserved
	Whether anyone may rightly prevent a ritual	12 to 13	Normative component of the thesis

Parent interviews covered the child's developmental history and diagnostic pathway, the cultural and religious practices the family held in relation to the child's condition, which practices had been attempted, what the family understood the benefits and harms to have been, and what the participant thought should be done where a practice proved harmful. Clinician interviews covered the diagnostic process, parental response to diagnosis, awareness of alternative treatments, disclosure by families, and views on responsibility for protecting children from harmful treatments. Focus group schedules opened with the explanation of ASD described above and moved to frequency of consultation, attributed causes, treatments advised, and perceived benefit and harm.

Interviews were conducted in English, and participants moved into isiZulu or Sesotho where they preferred. Such switching occurred in a minority of interviews. The first author is a home-language isiZulu speaker. The research assistant, a psychology master's graduate employed for her research experience, is a home-language Sesotho speaker fluent in the other languages used, and she supported interviewing in both languages. Translation occurred at the transcription stage. Recordings of the individual interviews were transcribed by a qualified external transcriber, and passages spoken in isiZulu or Sesotho were translated into English by the research assistant, who also translated and transcribed the focus group recordings. The first author checked every transcript against the recording, giving particular attention to translated passages, and the quotations reproduced in this article were checked by her against the audio; for passages originally in isiZulu this check was made by a first-language speaker, and for passages originally in Sesotho it rested on the research assistant's translation. Culturally specific terms were not translated. Terms such as *imbiza, isihlambezo, ukufutha, umsamo, amasiko, ukubhula* and *uhlanya* were retained in the original in the transcripts and in all coding and reporting, and are glossed at first use in this article, since each carries meaning that an English equivalent would flatten (24). No back-translation was performed and no independent translator reviewed the translated passages; we record this as a limitation in Section 4.4. Focus groups ran for between two and three hours and were audio recorded with the permission of all members. The first author (AS) kept a reflexive journal throughout, recording interaction dynamics that the recordings did not capture, including the gendered patterns of turn-taking observed in the traditional healer group.

### 2.5 Analysis

#### 2.5.1 First round: cohort-level reflexive thematic analysis

The analysis proceeded in two distinct rounds, and we describe them separately. The first round was conducted for the doctoral study. Transcripts were analyzed thematically following the six phases described by Braun and Clarke (25), applied in the reflexive orientation that the same authors have since articulated more fully (26, 27): a single analyst coded the data and treated coding as an organic and iterative process rather than a reliability exercise, and no codebook was fixed in advance. Coding in this round was inductive. Codes were generated from the data at semantic and latent levels without reference to a predetermined framework, and the research questions guided which material was coded rather than how it was labeled. The first author read and re-read each transcript, generated initial codes across the corpus, collated codes into candidate themes, reviewed and refined those themes against the coded extracts, defined and named the final themes and produced the written analysis. Coding was done manually, by highlighting and annotating the transcripts, and no qualitative data analysis software was used. The full sequence was repeated approximately two weeks after the first pass to check for over-reading and to test the stability of the theme structure. Coding was organized by cohort, producing separate theme sets for parents, clinicians, traditional healers and religious leaders. Not all coded material was carried into the themes reported here. Material addressing the normative items in the guides was coded and reserved for the normative component of the thesis, as described in Section 2.5.3.

#### 2.5.2 Second round: deductive recoding into a route-of-administration taxonomy

For the present article a second round of analysis was undertaken on the coded data from the first round. Its purpose was descriptive classification rather than new thematic interpretation. Reflexive thematic analysis does not of itself generate a classification of practices, and we do not claim that it did. The taxonomy in Table 3 was produced by a deductive recoding step in which a framework of routes of administration, taken from pharmacological convention, was applied to the practices that participants had named, with categories added where the framework did not fit the data.

**Table 3:** Traditional and religious practices reported in relation to children with ASD, by route of administration.

Route	Practice	Reported by
Ingested	Imbiza (multi-ingredient herbal decoction)	Parents, healers
	Isihlambezo (herbal tonic)	Healers
	Undisclosed herbal solutions and concoctions (muthi)	Parents, healers, clinicians
	Boiled lizard water	Parents
	Church-supplied tea	Parents
	Blessed or holy water	Parents, clinicians
	Consecrated soil taken orally or in bathwater	Parents
Rectal	Enema (herbal preparation, volume set by practitioner)	Parents, healers, clinicians
Inhaled or applied by vapor	Incense burning	Parents, healers
	Ukufutha (steaming under a blanket)	Parents
	Burning of substances and animal-derived material	Parents
Topical or applied	Anointing oils rubbed on the child	Parents
	Ropes or strings tied around the waist	Parents
	Incisions and cuts	Parents
	Bathing in prepared solutions	Parents
Proposed surgical	Cutting beneath the tongue to release speech	Parents, clinicians
Ritual and devotional, no substance	Prayer, in person and by television	Parents, religious leaders
	Deliverance and casting out of spells	Parents, religious leaders
	Laying on of hands	Religious leaders
	Calling of clan names at a designated site	Parents
	Graveyard visits	Parents
	Amasiko, family rituals of appeasement	Parents, healers
	Retrospective lobola and marriage rites for deceased kin	Healers
Diagnostic procedures	Prophecy, to establish cause	Parents, religious leaders
	Ukubhula, divination to establish cause	Healers

The procedure was as follows. The unit of analysis was the practice entry, defined as a discrete substance, procedure or ritual that any participant described as having been applied to, prescribed for or proposed for a child in relation to the condition. The second author (CM) read every coded extract from all four cohorts and recorded each practice entry together with the participant identifier and cohort, the route of administration where the participant had described one and any detail given on composition, dose, provenance or the person who administered it. The initial framework comprised four routes: inhaled, ingested, rectal and topical or applied. Categories were then added inductively where reported practices did not fit it. Ritual and devotional practices involving no substance formed the largest of these. Diagnostic procedures that determine which treatment follows formed another, and the proposed surgical procedure described in Section 3.6 was given a category of its own because it is unlike any other practice reported. Where the same practice was described under different names, entries were merged only where the descriptions agreed on both substance and route and were otherwise kept separate; undisclosed herbal preparations described only as *muthi* were pooled into a single entry because no participant could distinguish between them. Where different participants described the same practice inconsistently, as with *imbiza*, which practitioners said is given orally or rectally, the practice was recorded once with both routes entered, placed in the table under the route most often described, with the alternative recorded in the text. Where a single interview yielded several practices, each was recorded as a separate entry under the same identifier, so the table reports the cohorts that named a practice rather than the number of participants, and no practice was counted more than once per participant. Route was assigned only where a participant had described how the substance entered the child's body. Where no route was described, the entry was classified by the practitioners' account of usual administration if one existed, and otherwise recorded as undisclosed. Recoding was done manually in a spreadsheet, and every entry was linked to its source extract so that each classification could be traced back to the words of a participant.

The recoding was then checked against the transcripts by the first author (AS), who had conducted the interviews and the first-round analysis. Disagreements were resolved by returning to the extract together and, where the extract did not settle the matter, by following the parent's description, since parents had observed the administration. Two further rules were applied to guard against distortion of participants' meaning. Participants' own terms were retained as the name of each entry wherever one was given, in isiZulu where that was the language used. Extracts were kept verbatim and reread in context before each classification decision, and classification recorded only how the practice entered the body and who reported it, making no judgement about efficacy or risk; that judgement is confined to the Discussion and is identified there as ours.

#### 2.5.3 Scope of the present analysis and material excluded

The interview guides contained a substantial number of items on societal protection and ethical obligation (Table 2). These items served the second, normative objective of the doctoral study, which was to articulate and defend the ethical responsibilities arising from the empirical findings, and the material they generated was analyzed in that component of the thesis (20) rather than here. The present article is confined to the empirical objective. The criterion for inclusion in the Results was that an extract named, described or appraised a specific practice applied to or proposed for a child, or described a condition of its administration, namely dosing, composition, disclosure to clinicians or the training of the person administering it. Extracts in which participants offered normative views, on who ought to protect children or on the rightness of a practice, were excluded from the Results. Where such material bears on the regulatory context of the practices it is drawn on in the Discussion, and it is identified there as participant opinion rather than as a finding about practice. This boundary was deliberate, but it does mean that participants' own views on protection and responsibility are under-represented in this article relative to their weight in the interviews, and we record this in Section 4.4.

#### 2.5.4 Use of the published literature

The third objective involved reading the reported exposures in the light of published evidence of harm. We did not conduct a systematic review for this purpose, and we make no claim to have identified or appraised the harm literature systematically. The sources drawn on in Section 4.2.3 were identified through targeted searches of PubMed and Google Scholar for clinical reports of harm from traditional treatments administered to children in Southern Africa, supplemented by the reference lists of those reports and by the literature reviewed for the doctoral study. The mapping of that literature to individual practices is a contextual interpretation offered by the authors, and we have framed it as such throughout.

Extracts are reported verbatim. Punctuation and obvious transcription slips have been corrected silently, omitted material is marked with an ellipsis, and inserted clarification appears in square brackets. Non-contiguous passages have not been joined. IsiZulu terms are retained and glossed at first use. Parents and caregivers are identified as P1 to P24 and clinicians as HP1 to HP10. Focus group extracts are attributed to the group rather than to named individuals, since the recordings did not permit reliable individual attribution across a group discussion; each extract represents a single speaker. Where an extract referred to a living person by name or initials, the identifier has been removed.

### 2.6 Trustworthiness and reflexivity

Rigor was addressed using the criteria set out by Lincoln and Guba (28). Credibility was supported by data source triangulation across the four cohorts and the three collection methods (29). We use triangulation in its comparative sense. Setting accounts given from different positions alongside one another allowed us to identify where they converged and where they diverged, and divergence was treated as informative in its own right rather than as a failure of one account to confirm another. No account was taken to verify another. Credibility was further supported by verbatim reporting of participant accounts and by the freedom of participants to withdraw at any point, which none did. Member checking was not undertaken; participants did not review transcripts or analytic outputs, and we discuss the implications in Section 4.4 (30). Dependability was supported by supervisory review of the data and the analytic process and, for the present article, by the first author's independent checking of the recoding described in Section 2.5.2. Confirmability was supported by the reflexive journal, which the first author used to record and interrogate her own assumptions during collection and analysis. Transferability is addressed in the limitations.

The first author is a Black South African woman, a home-language isiZulu speaker raised in KwaZulu-Natal, a psychology graduate, and the mother of a son diagnosed with ASD at the age of four (at the time of data collection). Her proximity to the subject shaped the study in ways that cut in both directions. It gave her access to the emotional register of the parent interviews and made the accounts intelligible in a way that a researcher without that history would have had to work to reach. It also created a standing risk that she would hear her own experience in the data. The reflexive journal was kept for this reason. She was also read by the traditional healer participants as a representative of the university and therefore of a knowledge system that they described as having historically extracted from theirs, and this affected what they were willing to disclose about the composition of their preparations. We report that reluctance as data rather than treating it as missing information.

No patient and public involvement group was convened to co-design the study, and no advisory group of parents or practitioners reviewed the interview guides or the analysis. The design was shaped in two other ways. The first author's standing as a parent of a child with ASD informed the framing of the parent guide and the conduct of those interviews, and the two practitioner organizations set the conditions of their members' participation, including the requirement for a briefing on ASD before discussion. Neither amounts to involvement in the sense described in current guidance (31). We identify the absence of structured involvement as a limitation of the study and as a priority for any further work on this question, where a parent advisory group and practitioner representation at the design stage would strengthen both the instruments and the interpretation.

### 2.7 Ethics

The study was approved by the Human Research Ethics Committee (Non-Medical) of the University of the Witwatersrand, clearance number H17/11/52. Approval was granted on 17 November 2017 and was valid until 12 December 2020, and all data were collected within this approval period. Approval covered the participant information sheet, the consent forms and all three interview guides.

All participants received written information about the study and gave written informed consent before participation. Focus group members signed an additional undertaking to keep the content of the discussion confidential, having been informed that anonymity within the group could not be guaranteed. Identifying information relating to participants and to their children was removed at transcription. Participants could withdraw at any point without consequence.

Traditional health practitioners and religious leaders were reimbursed for travel and provided with refreshments in accordance with the protocol their organizations apply to all researchers. These costs were anticipated in the approved protocol and were met by the first author. Because parents of children with ASD report elevated parenting stress, arrangements were in place to refer any participant requiring psychological support to the Wits Emthonjeni Center.

The analysis reported here is a secondary analysis of data collected under the above clearance for the purpose of examining traditional and religious treatments and their effects on children with ASD. This purpose falls within the scope of the original consent, which provided for retention of the data and its use in subsequent journal articles.

### 2.8 Terminology

We use “children with ASD” throughout for consistency with the DSM-5 nomenclature under which the participating children were diagnosed and under which the practitioner briefings were framed (23). We recognize that identity-first language is preferred by much of the autistic community and by a growing share of the literature, and our usage should not be read as a position on that question. We use “traditional health practitioner” for practitioners falling within the categories defined by the Traditional Health Practitioners Act 22 of 2007 (19), and “religious leader” for pastors, prophets and reverends offering faith-based healing. Where participants used isiZulu terms we retain them and gloss them at first use.

## 3 Results

### 3.1 The treatment landscape

Every child in this sample had received medical care, since parents were recruited from hospital services. Every child had also received at least one traditional or religious practice directed at the condition, and most had received several, in sequences that ran alongside hospital treatment rather than replacing it outright. Parents did not describe choosing between systems. They described moving between them, often returning to whichever had most recently offered an account of cause.

Twenty-five distinct practices were named across the four cohorts, of which twenty-three were therapeutic and two were diagnostic. Table 3 sets them out by route of administration. The classification is ours and no participant used it, but it is the organization that carries most clinical meaning, since the risk attaching to a practice follows from how a substance enters a child's body rather than from whether the practice is described as traditional or religious. Several practices were named by more than one cohort, and we mark this in the table because convergence across cohorts, and divergence where accounts differed, is informative about how widely a practice is recognized. It does not verify any individual account. Entries attributed to traditional health practitioners and religious leaders are retrospective accounts of what those practitioners administer to children they recognized from the DSM-5 description given before the discussion, rather than records of treatment given to children with a confirmed diagnosis. Entries attributed to parents and clinicians concern children with a hospital-confirmed diagnosis.

Prophecy and *ukubhula* are diagnostic rather than therapeutic. We include them because in both practitioner systems the diagnostic procedure determines which treatment follows, so a treatment cannot be understood apart from the reasoning that selected it.

### 3.2 Ingested preparations

Ingested preparations were the most frequently reported category and the least specified. Parents could describe what they were told to do with a substance and could rarely describe what the substance was.

*Yes, they gave him muthi that I bath him with and also give him to drink* (P4).

*I took him to ZCC [Zion Christian Church] and they gave us the tea but after drinking the tea, there were no changes in the child's condition* (P12).

One account describes a preparation made from a live animal, given to the child to drink, with instructions governing its disposal:

*That man said we must go and find a big lizard. Then, we found that lizard; he said we must boil it; I don't know what he did but he boiled it until it died; the child drank that water; he said I should go throw away the lizard in a place a little far like deep Soweto and never look back after throwing it away* (P8).

Traditional health practitioners named *imbiza* and *isihlambezo* as the principal preparations they would give to a child presenting in the way described to them. *Imbiza* is a compound decoction of roots, leaves, bulbs and rhizomes, administered orally or rectally at a dose set by the practitioner according to the presentation of the child. Practitioners stated that pediatric and adult doses differ, and did not state that the constituents differ.

Practitioners declined to name constituents. This was a settled position across the group rather than an individual reservation, and the stated reason was prior extraction of indigenous knowledge by researchers and clinicians:

*That is why even now we are still fighting it, we are still trying to get the western and African to sit down and see because they come with a mindset that they know but they don't know anything, they come with a mindset of making us blind and use big words... but that was their way of taking our land right, they took our land, took our gold, took our minerals, it was their way* (Traditional healer, focus group).

Clinicians described the same information gap from the receiving end:

*It is difficult for us doctors to evaluate because we don't know what the child is going to be given* (HP3).

*We would explain that to the caregiver and say please, by all means, go to the traditional healer but should they suggest that there is something that you give your child, maybe, before you give that to your child, discuss that with your medical professional, in terms of the safety of giving it to a child. When I asked mom about what was in the water she said she didn't know what was in the water* (HP1).

The composition of ingested preparations was therefore unknown to the parent administering them, unknown to the clinician treating the child, and withheld by the practitioner supplying them. No participant in any cohort could state what a single child had ingested.

### 3.3 Rectal administration

Enemas were reported by parents, described as standard practice by traditional health practitioners, and identified by clinicians as a route of presentation to the hospital. The preparation is typically *imbiza*, introduced rectally at a volume determined by the practitioner.

Parents raised safety concerns about this route without prompting, and framed them in terms of the training of the practitioner rather than the practice itself:

*I think they will put a child in danger, you know why sister? So now you will take this child to that person and put the child in the steamer [ukufutha], cover the child with a blanket, and other kids may have seizures and all that, and the child will develop other illnesses, better I just follow this one at the hospital* (P5).

*I think they will harm the child worse, these people train in different ways. Others are trained with animals, others with trees, others they pray, you see? So my belief, is that I don't think they can help the child* (P9).

Clinicians reported children presenting acutely after traditional treatment, though they did not consistently attribute presentations to route:

*Here, in hospital general and not speaking about ASD just general we see children coming back intoxicated coming back who've been to traditional healers and have been exposed to traditional random things very ill* (HP8).

*Exposing children to alternatives, I think has a potential direct harm, where children are given certain substances* (HP3).

### 3.4 Inhaled preparations and steaming

Incense burning was reported by parents and also described by practitioners, who described it as part of the diagnostic procedure at *umsamo*, the sacred space in which the practitioner addresses the family ancestors, and as a means of clearing a dwelling of malign influence. *Ukufutha*, steaming a child beneath a blanket with a herbal preparation, was described by parents. The account at P5 above (see 3.3) links steaming to seizure risk, and this was a parent's own inference rather than clinical advice she had received.

### 3.5 Topical and applied practices

Parents described oils, consecrated soil, holy water, bathing preparations, and cords tied around the child's waist. Applied items were most often protective in purpose, intended to shield the child from an identified agent rather than to treat a symptom:

*So, they put ropes around his waist to protect him from whoever that's against the child* (P17).

*At church, they just prayed and gave him water, said we must give him to drink, sharp; they gave him soil, that we must put it on him on the head like that... when you bathe him you must put it in the water and bathe him in it, also, pour this water for him when he wants to drink, give him that water and sprinkle the yard with it* (P2).

*I just see them giving the child some oil; they rub the child with that oil. I don't see any difference. I just see her the same* (P1).

Incisions and cuts appeared in the parent accounts as a category of practice without the detailed description attaching to other routes.

### 3.6 A proposed procedure

One practice sits outside the therapeutic categories because it was proposed rather than performed, and because it is surgical. A prophet consulted by a family undertook to arrange the cutting of tissue beneath the child's tongue on the reasoning that this would release speech:

*The Zionist guy told us also during his visit at our house that he was going to find a guy that will cut something below the child's tongue so that he can talk* (P17).

The parent did not consent. The proposition was not isolated, and clinicians reported receiving the same request from families in the opposite direction:

*In my experience in this unit, I have come across parents who will say, please, check the child's tongue; maybe, it is stuck* (HP4).

We have given this practice its own category in Table 3, and its own section here, for two reasons. It involves cutting a child, which sets it apart from every other practice reported. It also shows a belief about cause translating directly into a surgical remedy, since the prophet's reasoning was that speech was mechanically obstructed.

### 3.7 Ritual and devotional practices

Prayer was the most widely used practice in the sample and the one most parents named first. It reached families through congregations, through home visits, and through television broadcast:

*There's a church that I watch on television called Emmanuel TV. Emmanuel TV helped me a lot because I didn't have a break. So, normally when I switch on Emmanuel TV and pray with her, she puts her hand; she actually sleeps you know* (P9).

Reported outcomes varied. A minority of parents described change:

*His behavior before prayer wasn't satisfactory; he would cry for no reason and there was no way to stop him; he would stop crying maybe after five to six hours crying but now he's calm; he's alright; he's sharp* (P10).

Most did not:

*We took her to some lady who is a prophet there, she said there was nothing she could do because when she looks at the child's condition; there is nothing she sees; she said she did not even believe that the child was bewitched* (P13).

*They just prayed for him... They said at a certain time, we must pray. There weren't a lot of changes; the crying would stop a little after prayer, then, again it will start* (P20).

Religious leaders held that healing capacity is unevenly distributed and that outcome depends on the standing and the specific gift of the person praying:

*You take somebody who's got the gift of tongues... this person doesn't function in healing, doesn't function in deliverance, is not called for that, so you can bring your child, they take the child to a pastor, the pastor prays for the child and it becomes like God doesn't hear. Only that the problem is not that God doesn't hear, God heard the prayer alright but the person that is not their platform* (Religious leader, focus group).

Parents were not aware of this distinction. None reported having asked whether a particular leader claimed a healing gift before bringing a child.

Family ritual, *amasiko*, was the second devotional category, and it was frequently prescribed by a church rather than by a traditional practitioner:

*At church they would say they must do amasiko from his father's home, you see things like that. They say the ancestors are fighting over him; it's a struggle, things like that* (P3).

Traditional practitioners located the origin of a child's condition in unresolved family history and prescribed accordingly, including rites performed on behalf of deceased kin:

*When you dig even more you find that the grandparents of this child have never been married, they were just cohabiting... In issues like this, we have to get those elder people married first, it is said that in most instances they are already in their graves. The process will start by just getting them first to pay the lobola and followed by a wedding ceremony. This is done because things are not going well for children... Nothing will change or improve until we find the truth of the problem, how it started* (Traditional healer, focus group).

### 3.8 Findings that cut across routes

Traditional practitioners, speaking of children they recognized from the briefing, rejected the term autism and offered no equivalent in any African language. Presentations consistent with ASD were classified within the existing category of *uhlanya*, glossed by participants as insanity, and described as a child who does not respond, does not answer, breaks what is given and does not follow instruction. One practitioner set out the consequence directly:

*The problem is that autism, only white people check it; we don't check it traditionally because if you bring the child with autism to me and I check them by way of traditional, I will find different answers and different causes so, I will not come to you and say it's autism* (Traditional healer, focus group).

Without a distinct term there is no distinct treatment protocol, and children with ASD receive whatever is indicated for the broader category into which they fall.

Dosing followed no standard, on practitioners' own account. Dosage was set by the practitioner on assessment, and no participant described a written protocol, a measured quantity or a rule for adjusting an adult preparation for a child. Practitioners stated in general terms that pediatric and adult doses differ, without describing how the difference is arrived at in any particular case.

Practitioners themselves raised the problem of unverifiable training. The most direct evidence came from within the traditional healer group, where one member challenged another's knowledge of a basic preparation in front of the group:

*But this one doesn't know, they only heard that it's Isihlambezo, they don't know whether it's the one to strengthen the womb after miscarriage or what?... Some, you can't give a person because it could make a child hyperactive... the baby keeps on rolling and can get to a point where it ties itself with the umbilical cord to point the baby dies* (Traditional healer, focus group).

The same group acknowledged that entry into practice is not controlled:

*With traditional healers, some are there today and gone tomorrow they've started these fly-by-night practices, just like there are also bogus doctors, there are also bogus traditional healers too* (Traditional healer, focus group).

Religious leaders made the corresponding admission about their own sector, including a first-hand account of a televised healing claim:

*I was with [a pastor] on TV the other day and he was claiming to cure TB... they wanted to confirm that [he] cured the baby's TB, the child was in ICU. I refused, I said TB is curable after six months* (Religious leader, focus group).

Both practitioner groups called for regulation of their own sector, and neither treated the problem as external to it.

Disclosure to clinicians was partial and ran in one direction. Parents shared some of what they had sought elsewhere and withheld the rest, and clinicians described learning of it late, usually when a ritual required the child to leave the ward:

*They wouldn't tell us; they don't go into details as to what happens or what they get given or what they get told but what they feel comfortable sharing with us, is that they consulted a healer and they were told they need to perform a ceremony so if the child is admitted that's when they would ask for permission that can you please release the patient, we need to perform a certain ritual* (HP5).

Clinicians reported managing concurrent administration by asking families to choose, since they could not assess interaction risk against an unknown preparation:

*We tell them that we cannot be treating the child with this medication and then the next thing they'll be giving the traditional, so bombarding the child with all this medication it's quite harmful so they need to decide what is it that they want to do, should we stop our medication while they seek treatment traditional* (HP5).

Some families left the service altogether after diagnosis. Clinicians described parents who did not return once a diagnosis and a treatment plan had been given:

*We have had parents that do not come back after a diagnosis and a planned program. They say I will deal with this my own way* (HP4).

Clinicians linked this to the length of the diagnostic pathway and to the absence of a pharmacological answer:

*There's a perception that sickness is something you go to the doctor; you get medication and you get better. They just don't understand. I think many of our parents just come to the doctor thinking the doctor will give a pill* (HP3).

### 3.9 What families reported gaining

Parents did not report symptomatic improvement in their children from these practices, with the small number of exceptions quoted in 3.7. They did report gains of a different kind, and these were consistent enough across interviews to constitute findings in their own right.

The first was an account of cause. ASD was presented to families as a condition of unknown etiology, and traditional and religious explanation supplied what the clinical account withheld. The second was continuity with family and community, since consultation drew in grandparents, in-laws and elders and located the child within a kinship structure rather than outside it. The third was a route by which action remained possible after the clinical pathway had reported that no cure exists. Where one practice failed, another remained available, and parents described sustained searching rather than resignation. One parent had traveled through all nine provinces.

These reported benefits attach to the parents and to the family. No participant in any cohort described a benefit accruing to the child from the practices themselves, other than the settling reported at P9 and P10 (see 3.7).

## 4 Discussion

The discussion is set out in three parts, followed by the limitations. Section 4.1 restates what participants reported, without inference. Section 4.2 gives our interpretation of those findings, including our reading of the reported exposures against the published literature, and is identified throughout as interpretation. Section 4.3 sets out recommendations, which are confined to the South African setting in which the data were collected and rest on the findings reported in Section 3 and on the literature cited.

### 4.1 Empirical findings

Every child in this sample had received at least one traditional or religious practice directed at the condition, most had received several, and these ran alongside hospital care rather than replacing it. Twenty-five distinct practices were named across the four cohorts. They fell into six categories by route of administration, set out in Table 3, with one proposed surgical procedure standing apart. No participant in any cohort could state the composition of any ingested or rectally administered preparation. Parents did not know what they had given and clinicians reported that they were not told. Practitioners declined to disclose and gave as their reason a history of extraction of indigenous knowledge. Practitioners stated that dosing was set on assessment of the individual child and that pediatric and adult doses differ; no participant described a written protocol. Practitioner participants had no term for the presentation described to them and placed it within the category of *uhlanya*. Parents described disclosing some of what they had sought elsewhere to clinicians and withholding the rest, and clinicians described responding to concurrent use by asking families to choose between systems. Some families did not return after diagnosis. Both practitioner groups said that entry into their sectors was uncontrolled and called for regulation. Parents reported that the practices had given them an account of cause and had kept the wider family involved and hope alive, and, with two exceptions, they did not report symptomatic change in their children.

### 4.2 Interpretation

#### 4.2.1 Specification as the prior problem

The usual conclusion drawn about traditional treatments for ASD is that the evidence supporting them is weak. Our reading of these data is different and more specific. For the preparations most likely to cause harm there is no evidence, and there is at present no mechanism by which evidence could be generated, because the intervention has no stable definition. An unnamed preparation, compounded to no formula, dosed by clinical impression and supplied by a practitioner whose training cannot be verified, is not a candidate for appraisal. It is a category of exposure rather than a treatment.

This matters for any assessment of the quality of the evidence base, because it identifies the floor. Appraisal presupposes a specified intervention. Where the specification is absent, the prior task is to make appraisal possible, and that is a regulatory task before it is a scientific one.

#### 4.2.2 Where these practices sit relative to the appraised interventions

The conventional framing places evidenced medicine on one side and unevidenced healing on the other. On close reading, the ASD intervention literature does not sustain that division.

The interventions with the strongest support in ASD are behavioral and developmental. The most recent synthesis of that literature reports modest effects, with significant summary estimates from randomized trials for developmental interventions on social communication and for naturalistic developmental behavioral interventions on adaptive behavior, language, play and social communication, and with effects attenuating when caregiver-report outcomes are excluded (2). The same synthesis records that potential benefits cannot be weighed against potential adverse effects, because adverse effects are inadequately monitored and reported across the field. Pharmacotherapy sits in a comparable position. Two agents carry regulatory approval in the United States, both for irritability, which is a comorbid presentation rather than a core feature.

Harm is therefore appraised poorly across the field, and what we describe is a severe instance of a general weakness rather than a foreign problem. The difference is one of degree, and the degree is decisive. For behavioral interventions the intervention is specified and the harm monitoring is deficient. For the preparations described here, neither the specification nor the monitoring exists. A trial of *imbiza* for ASD could not be designed today, because no two practitioners would be administering the same thing.

This bears on how our argument should be read. We are not asking traditional treatments to meet a standard that biomedical interventions themselves fail. The more useful point is that the case for harm reporting in ASD care is a general one, and a mechanism built to capture adverse events from traditional preparations would serve the appraised interventions too.

#### 4.2.3 Risk by route of administration: a contextual interpretation

What follows is our interpretation of the reported exposures in the light of published clinical literature. As set out in Section 2.5.4, that literature was identified through targeted rather than systematic searching, and the mapping of risk to route is a reading offered by the authors rather than an appraisal of each practice against a defined body of evidence. We present it so that the reasoning behind the route-based recommendations in Section 4.3 is open to scrutiny. Route of administration turns out, in our reading, to be the more useful organizing principle. Sorted this way, the practices reported here separate into a small number that carry serious documented risk and a larger number that carry little or none, and the division does not follow the line between traditional and religious healing.

Rectal administration is the route for which harm to Southern African children is best documented. A South African case series described a distinct clinical picture in children admitted after traditional enema administration, with deterioration within twenty-four hours in most cases, and in-hospital mortality of twenty-eight per cent, rising to forty-three per cent where the enema was herbal rather than chemical (14). Documented mechanisms include rectal perforation, enema-induced colitis progressing to necrosis of the bowel wall, and electrolyte disturbance, and reported constituents have included nicotine and potassium dichromate (32). An earlier southern African admission series found traditional medicines to be the second commonest cause of acute poisoning admissions and the largest single contributor to poisoning deaths, at a case fatality several times that of the commonest exposure, with roughly one in ten of those exposures administered rectally (13). A recent case series from a Johannesburg pediatric surgical service describes young children requiring major operations for colonic injury after traditional enemas (15), which indicates that the picture characterized in the 1990s persists.

Most of these findings date from the 1980s and 1990s, and their age is itself informative. The clinical harm of pediatric traditional enema use was characterized decades ago, and the practice continues, which suggests that the failure has been one of regulation rather than of knowledge.

Ingested preparations carry an unquantifiable risk and one specific quantifiable one. The unquantifiable risk follows from unknown composition. The specific risk arises where a child is also prescribed medication, since neither the clinician nor the parent can assess interaction against an undisclosed compound. Clinicians in this study resolved that impasse by asking families to choose between the two, which removes the child from prescribed treatment for the duration of the traditional course. The clinical reasoning is sound and the outcome is adverse, and it follows directly from non-disclosure rather than from any decision either party wished to make.

Inhaled preparations and steaming carry, in our reading, a population-specific risk. Epilepsy is among the most frequent comorbidities in ASD. Meta-analytic estimates place its prevalence in autistic populations well above the general population figure of about one per cent, and higher again where intellectual disability co-occurs (33). One parent in this sample first sought help after her child had seizures at five months. A parent identified the risk herself, describing steaming beneath a blanket as dangerous for a child who might seize. Hyperthermia and airway compromise in an enclosed space are foreseeable in a young child who may be unable to signal distress, and children with limited expressive speech are less able to signal it than others.

Topical and applied practices carry lower but non-trivial risk. Cords tied around the waist of a child present a ligature hazard. Incisions performed outside sterile conditions present an infection hazard. Neither is documented in this population; both are plausible, and both are inferred rather than observed.

The proposed lingual procedure sits in a category of its own. A non-clinician undertook to arrange the cutting of tissue beneath a child's tongue on a theory that speech was mechanically obstructed. The parent declined. Clinicians reported families asking them to check the same thing, which shows the theory circulating rather than sitting with one practitioner. Frenotomy performed by an unqualified person on a child who cannot report pain or complication is, in our judgement, the most serious single risk in these data, and it is the one for which the regulatory gap is most stark, since no category of registration contemplates it.

Devotional practices involving no substance carry no direct physical risk. Prayer, prophecy, laying on of hands and the calling of clan names are physically inert. Any harm they carry is indirect, through delay, through financial extraction, and through explanations that attribute the child's condition to parental conduct or to malice. Religious leaders in this study described the commercialization of healing within their own sector with some candor, including payment expectations attached to prayer.

This distribution has a practical consequence. Regulating by route separates a small number of high-risk practices from a large number of low-risk ones, and it allows a proportionate response where a blanket position on traditional and religious healing cannot deliver one.

#### 4.2.4 The reported benefits, and who receives them

Parents described three gains: an account of cause where the clinical account offered none, continuity with family and community, and a preserved capacity to act after being told that no cure exists. These should not be dismissed. Caregivers of children with ASD report elevated stress, depression and anxiety relative to other parent populations, and their psychological state is a legitimate clinical target in its own right. Parent-focused interventions improve caregiver mental health, and parenting interventions promoting responsiveness show the largest effects on the parent-child relationship (34). A need that traditional and religious consultation is currently meeting is therefore a need that clinical services could meet directly.

The finding that constrains this argument is that no participant in any cohort described a benefit accruing to the child from the practices themselves, beyond two accounts of a child settling. The beneficiary of the reported gains and the bearer of the physical risk are therefore different people.

That asymmetry sets a limit on how far cultural respect can carry an argument about exposure. A parent's need for an account of cause is real and deserves a response. It does not license the administration of an unknown compound to a child by an unverifiable route. The proper response to the need is to meet it directly, through explanation, through post-diagnostic support and through community education, rather than to tolerate a risk to the child as the price of meeting it.

#### 4.2.5 Delay and disengagement

Clinicians reported families who did not return after diagnosis. Set against evidence that developmental and naturalistic developmental behavioral interventions produce measurable gains in social communication and adaptive behavior in young children, disengagement carries a real cost, even allowing for the modest size of those effects (2).

Attributing that delay to families would misread these data. Parents described referral from clinic to clinic, waits of up to three years for assessment, diagnostic intervals of six months at the receiving hospital, and district facilities without the capacity to screen beyond speech therapy. Several consulted traditional and religious practitioners before reaching the hospital, and did so because the hospital pathway was slow, opaque and offered no account of cause. The two systems are competing on responsiveness, and the public system is losing on it.

This reframes the responsibility. A health system that cannot deliver a timely diagnosis, and cannot explain etiology, and cannot offer post-diagnostic support, generates the conditions in which families seek explanation elsewhere. Reducing exposure to harmful practices therefore requires investment in the clinical pathway alongside regulation of the practitioners.

#### 4.2.6 The regulatory position

The Traditional Health Practitioners Act 22 of 2007 was enacted to provide a regulatory framework for the safety, quality and efficacy of traditional health care and to control registration, training and conduct (19). Draft regulations setting out registration processes, categories, forms and fees were published for comment in June 2024 (35).

The Act is still not operative. In May 2026 the Portfolio Committee on Health received a briefing from the Council and found that the regulations required to bring the Act into operation remain outstanding, that no functional national register of traditional health practitioners exists and that enforcement mechanisms are absent (5). The Council and the Department attributed the delays to the absence of promulgated regulations, to funding and capacity constraints, and to outstanding procurement of registration systems, and reported that an amendment Bill is in process to convert the Council from an interim to a permanent body, and that a national traditional medicine policy aligned to World Health Organization strategy is at an advanced stage.

The scale of the mismatch is worth stating in figures. The committee questioned how the Council is expected to regulate an estimated 300,000 practitioners nationally on an allocation of approximately R6.7 million, with projections below R10 million in subsequent years, and questioned a funding model that expects the Council to sustain itself on registration fees drawn from practitioners working in economically marginalized and rural communities (5).

Nineteen years after enactment, therefore, a practitioner cannot be verified as registered, because there is no register to consult, and cannot be sanctioned, because there is no mechanism to sanction through. This is the finding of a parliamentary committee rather than an inference of ours, and it is consistent with what practitioners in this study said about their own sector, including the acknowledgment of untrained members within the group.

The international policy position has meanwhile moved. The Global Traditional Medicine Strategy 2025 to 2034, adopted by the Seventy-eighth World Health Assembly, sets four objectives, among them strengthening the evidence base and ensuring safety and regulation, and it identifies the misappropriation of traditional knowledge as a live obstacle to cooperation (17). South Africa has a policy commitment consistent with that strategy (18) and no operative machinery to deliver it.

### 4.3 Recommendations: what regulated integration would require in South Africa

Integration is often proposed in general terms and rarely specified. The recommendations that follow are confined to the South African setting in which the data were collected. They rest on the findings reported in Section 3 and on the published literature cited, and we make no claim that they transfer to other African countries, whose practitioner sectors and regulatory arrangements differ from South Africa's and have not been studied here. Within that boundary, our data support six requirements, sequenced by what they cost and by how quickly they could be implemented.

The first is routine, penalty-free enquiry at the clinical encounter. Non-disclosure was near universal here and it is the proximate cause of the interaction problem described in Section 4.2.3. Asking every family, recording the answer, and responding without censure would surface exposure that is currently invisible. This costs nothing and requires no legislation.

The second is advice framed by route rather than by system. Clinicians in this study did not know where to draw a line and defaulted to either blanket respect or a forced choice. Route-based guidance gives them a defensible position: devotional practice involving no substance raises no medicines-safety question, and any preparation entering the body by mouth or rectum requires disclosure of contents before administration to a child.

The third is a name for the condition, and basic biomedical orientation. Practitioners had no term for ASD and classified it within a category glossed as insanity, so children with ASD receive whatever that category indicates. Terminology development with practitioner bodies is a low-cost intervention with disproportionate reach, and it is a precondition for any condition-specific guidance. It also has a receptive audience. South African practitioners have themselves identified biomedical knowledge and skills as a training need (6), and both groups in this study accepted a briefing on ASD as a condition of participation.

The fourth is constituent disclosure with intellectual property protection. Practitioners withheld composition because of a documented history of extraction, and treating that refusal as obstruction would guarantee it continues. Disclosure to a protected register, with enforceable rights over the knowledge disclosed, addresses the stated reason for refusal. The WHO strategy names this problem directly (17), which gives a South African scheme an international frame to work within.

The fifth is a functional register with enforcement. This is the item that requires the state to act, and it is the item on which the parliamentary record shows nineteen years of failure.

The sixth is an adverse event reporting pathway. Children presenting after traditional treatment are currently recorded, where they are recorded at all, under general poisoning codes, as in the admission data from the study hospital itself (36). A specific reporting route would convert clinical experience into the evidence base whose absence this article documents, and would allow the risk of individual practices to be quantified rather than inferred.

The two practitioner groups in this study asked for regulation of their own sectors. In this setting, at least, the obstacle does not appear to be practitioner resistance.

### 4.4 Limitations

Participants were recruited from a single urban tertiary hospital, and the sample was entirely Black African and predominantly composed of mothers and grandmothers. Practices in rural settings, in other language communities and among other population groups are not represented, and the reported gender pattern reflects who attends clinics with children rather than who makes decisions in families. The findings describe one urban tertiary setting in one province, and we do not extrapolate them to other settings in South Africa or to other African countries.

The most consequential limitation is one of selection. Parents were recruited inside the health system, so families who disengaged entirely are absent from the sample by construction. Clinicians described such families. If exposure to unmonitored practices is heavier among them, and it is reasonable to expect that it is, the harms reported here understate the true position.

Severity of presentation was not systematically recorded, so we cannot examine whether treatment-seeking varied with the child's presentation. No outcome was clinically verified and no preparation was chemically analyzed, so all statements about effect and about harm rest on participant report or on the published literature. The refusal of practitioners to disclose constituents means that the central gap in these data is structural rather than accidental, and that gap is a finding as well as a limitation.

The data were collected before the current regulatory developments, and we have addressed that by testing the regulatory claims against the parliamentary record rather than against the position at the time of collection.

Four further limitations arise from the instruments and procedure. First, the interview guides devoted many items to societal protection and ethical obligation, and that material has been analyzed in the normative component of the thesis rather than here (Section 2.5.3). The practices reported in this article were therefore selected from a wider body of talk, and participants' own views on responsibility and protection are under-represented relative to their weight in the interviews. This is a selection effect of the article's scope rather than of the sampling, and it limits what the Results can say about how participants themselves weighed the practices they described. Second, practitioner participants were briefed on ASD before discussion. Their accounts are retrospective recollections of children they recognized from that description, which introduces the recall and response bias set out in Section 2.3. They should be read as evidence of what is administered to children presenting with these features; they are not evidence about children with a confirmed diagnosis, and the frequency with which practitioners said they see such children should not be read at all. Third, no member checking was undertaken. Participants did not review their transcripts or the analytic outputs, so the interpretations are ours alone and participants had no opportunity to correct or contest them. Member checking presents its own difficulties in a design of this kind, where focus group extracts cannot be attributed to individuals and where returning practitioner data through an organization could compromise members' confidentiality (30), but its absence remains a limitation. Fourth, interviews were conducted in English with switching into isiZulu or Sesotho, translated passages were rendered into English at transcription by a single bilingual research assistant, and no back-translation or independent review of the translations was carried out. Although culturally specific terms were retained and the first author checked the isiZulu passages, some loss of meaning in the translated passages cannot be excluded.

The demographic form collected limited information (Supplementary File 2). It recorded the child's sex and population group and the date of diagnosis, and it did not record the age, education, employment or home language of parents and caregivers, nor the age, sex or years in practice of clinicians and practitioners. The sample is therefore described in Table 1 by role and setting rather than by a full demographic profile, and we cannot examine whether the practices reported varied with those characteristics. Refusals among parents and clinicians were not systematically recorded, so we cannot characterize those who declined.

## 5 Conclusion

Every child in this Soweto sample had been given traditional or religious treatment alongside hospital care, and no part of that exposure was recorded or monitored in the health system. Among the practices reported, those most likely to cause harm are few and identifiable, and in our reading they are distinguished by route of administration rather than by belief system. The benefits families described accrue to parents rather than to children.

The evidence base for these practices cannot be improved while the interventions remain unspecified and the practitioners unregistered. Nineteen years after the enabling Act, South Africa has neither a register nor an enforcement mechanism, while both practitioner groups in this study asked to be regulated. Route-based risk triage, penalty-free disclosure at the clinical encounter, and constituent disclosure under protected intellectual property offer a proportionate starting point for South Africa, and each is achievable ahead of the wider legislative reform on which children are currently waiting. Whether the same pattern holds elsewhere in South Africa or beyond it is a question for further research, and we make no claim that it does.

## Data Availability

The raw data supporting the conclusions of this article will be made available by the authors, without undue reservation.

## Supplementary material

Supplementary Figure 1 Completed COREQ 32-item checklist.

Supplementary Figure 2 Demographic information sheets (participant and child).

Supplementary Figure 3 Semi-structured interview guides for parents, clinicians and focus groups.
