# Supplementary material for: Traditional and religious treatments administered to children with autism spectrum disorder in South Africa: a qualitative study of practices, composition and risk
Source: Front Neurol. 2026 Oct 30;17:1946904. doi: 10.3389/fneur.2026.1946904 (PMC13759395; doi:10.3389/fneur.2026.1946904)
Supplement: Supplementary file 1 [file Data_Sheet_1.pdf]

Supplementary File 3

Semi-structured interview guides

Manuscript 1946904 (Simelane & Mapaling). The three guides are reproduced from the approved study protocol (Human Research Ethics Committee (Non-Medical), University of the Witwatersrand, H17/11/52). Items are numbered as in the original schedules; see Table 3 of the manuscript for the domains and where the resulting material is reported.

Guide 1: Parents and caregivers

1. Tell me about [the child and the family].
2. What cultural practices does the family believe concerning the child's condition?
3. Can you talk me through some of these practices?
4. Have you explored helping the child using this practice?
5. What were the benefits of this? (If 4 is yes)
6. Did it cause any harm? (If 4 is yes)
7. What was the reason for not exploring the practice? (If 4 is no)
8. What religious practices does the family believe in that can help the child?
9. Please describe these practices.
10. Have you explored helping the child using this practice?
11. What were the benefits? (If 10 is yes)
12. What were the harms? (If 10 is yes)
13. What was the reason for not exploring the practice? (If 10 is no)
14. What can society do to protect children if some of these practices (cultural or religious) prove to be harmful to the child?
15. What can be done to strengthen the societal obligation to the protection of children with ASD from cultural and religious practices?
16. What measures exist out there to protect children exposed to harmful cultural and religious practices?
17. What can society, in general, do to improve the wellbeing of children with ASD?
18. Can you think of interventions that can be implemented to improve the wellbeing of children with ASD?
19. If some of the practices are harmful, what can we do to protect children from harmful cultural practices and at the same time preserve our cultural beliefs?
20. In your opinion, what cultural practices are right to perform and which ones are not right?
21. How do you determine the rightness and the wrongness of a practice?
22. In those that are wrong or harmful, what can be done in that situation?
23. In the beneficial practices, what can be done to improve them?

Guide 2: Hospital clinicians

1. What is your involvement with children diagnosed with ASD?
2. Tell me about the process involved in diagnosing a child with ASD.
3. How much involvement is there between you and the parent/s?
4. In your experience, how do parents react when they are told about the child's condition?

5. In your experience and interaction with parents, have you come across parents who reject the child's diagnosis?
6. What would you say is the reason they reject the diagnosis?
7. What can you say about parents who seek alternative forms of treating their children with ASD?
8. Have you come across such parents who seek alternative forms of treatment?
9. What is usually the reason for them to seek other forms of medication?
10. Can you tell me what usually are these kinds of alternative medication?
11. Do you think it is right for parents to take their children to alternative forms of medication?
12. If 11 is yes or no, why? Tell me more.
13. What can you say about the issue of exposing children to other forms of medication, in terms of their wellbeing?
14. As a practitioner, can you share in your opinion who is obligated to protect children with ASD?

Guide 3: Focus groups with traditional health practitioners and with religious leaders

Interviewer instruction: explain the disorder comprehensively (structured explanation of ASD drawn from DSM-5 criteria, given before item 1).

1. Based on my explanation of autism spectrum disorders, have you treated a child with the condition I have explained?
2. Tell me how often you consult with these children.
3. Who usually brings the child?
4. What would you say is the cause of the disorder?
5. What do you usually tell parents is the cause of the disorder?
6. What do you advise parents on how the disorder can be treated?
7. Do you think the practice of cultural and religious rituals you perform can be beneficial to the wellbeing of the child?
8. If yes, how?
9. If no, why then are the practices of cultural and religious rituals performed?
10. Do you think the ritual may cause any form of harm to the child?
11. If yes, what form of harm? (i) What can you do to prevent this from happening? (ii) What can the parent do to protect the child?
12. Do you think it is fair or right for anyone to prevent this ritual from being performed?
13. If yes or no, expand, please.
