# Supplementary material for: Traditional and religious treatments administered to children with autism spectrum disorder in South Africa: a qualitative study of practices, composition and risk
Source: Front Neurol. 2026 Oct 30;17:1946904. doi: 10.3389/fneur.2026.1946904 (PMC13759395; doi:10.3389/fneur.2026.1946904)
Supplement: Supplementary file 2 [file Data_Sheet_2.pdf]

Supplementary File 2

Demographic information sheets

Manuscript 1946904 (Simelane & Mapaling). Reproduced from the approved study protocol (H17/11/52). Two sheets were used, one completed by the participant about the child and one recording the same fields for the child; the fields are identical and are listed once below.

Demographic information sheet: participant and child

Field	Response options
1. Child pseudonym	Free text
2. Gender	Free text
3. Nationality (population group and language or faith community)	African/Black: Zulu, Xhosa, Sotho, Setswana, Tsonga, Venda, Pedi, Swati, Ndebele, Other. White. Coloured. Indian: Hindu, Muslim, Christian, Other. Other.
4. Date of diagnosis	Free text
5. Has the child gone through any traditional related rituals because of his/her condition of ASD?	Yes / No
6. If yes, explain what type of rituals	Free text
7. Has the child gone through any religious related rituals because of his/her condition of ASD?	Yes / No
8. If yes, explain what type of rituals	Free text

Note. The sheet did not record the age, education, employment or home language of the adult participant, nor the age, sex or years of practice of clinicians, traditional health practitioners or religious leaders. See Sections 2.3 and 4.4 of the manuscript.
