# Supplementary material for: Traditional and religious treatments administered to children with autism spectrum disorder in South Africa: a qualitative study of practices, composition and risk
Source: Front Neurol. 2026 Oct 30;17:1946904. doi: 10.3389/fneur.2026.1946904 (PMC13759395; doi:10.3389/fneur.2026.1946904)
Supplement: Supplementary file 3 [file Data_Sheet_3.pdf]

Supplementary File 1

Consolidated Criteria for Reporting Qualitative Research (COREQ): 32-item checklist

Manuscript 1946904: Traditional and religious treatments administered to children with autism spectrum disorder in South Africa: A qualitative study of practices, composition and risk (Simelane & Mapaling). Checklist items follow Tong, Sainsbury and Craig (2007). Section numbers refer to the revised manuscript.

No.	Item	Guide question or description	Reported in (revised manuscript)
**Domain 1: Research team and reflexivity**			
Personal characteristics			
1	Interviewer or facilitator	Which author/s conducted the interview or focus group?	The first author (AS) conducted all individual interviews and facilitated both focus groups, supported by a research assistant (Section 2.4).
2	Credentials	What were the researcher's credentials?	AS: psychology master's graduate and doctoral candidate at the time of data collection (Sections 2.6, Acknowledgements). CM: Professor of Psychology and registered clinical psychologist (title page).
3	Occupation	What was their occupation at the time of the study?	AS: doctoral candidate, University of the Witwatersrand (Acknowledgements).
4	Gender	Was the researcher male or female?	Female (Section 2.6).
5	Experience and training	What experience or training did the researcher have?	Training in psychology; prior master's research with parents of children with ASD; research assistant held a master's degree in psychology and was employed for her research experience (Sections 2.4, 2.6).
Relationship with participants			
6	Relationship established	Was a relationship established prior to study commencement?	No prior relationship with parents or clinicians. Practitioner organisations were approached through gatekeepers and a briefing meeting preceded each focus group (Sections 2.3, 2.4).
7	Participant knowledge of the interviewer	What did the participants know about the researcher, e.g. personal goals, reasons for doing the research?	Participants received written information on the study and its aims (Section 2.7). Practitioner participants positioned the researcher as a representative of the university (Section 2.6). Parents were told that AS is herself the mother of a child with ASD.

No.	Item	Guide question or description	Reported in (revised manuscript)
8	Interviewer characteristics	What characteristics were reported about the interviewer/facilitator?	Black South African woman, home-language isiZulu speaker raised in KwaZulu-Natal, psychology graduate, mother of a son diagnosed with ASD; the effects of this proximity on access and on disclosure are discussed (Section 2.6).
**Domain 2: Study design**			
Theoretical framework			
9	Methodological orientation and theory	What methodological orientation was stated to underpin the study?	Qualitative, exploratory and descriptive design; reflexive thematic analysis in the first round, deductive classification by route of administration in the second (Sections 2.1, 2.5.1, 2.5.2).
Participant selection			
10	Sampling	How were participants selected?	Non-probability purposive sampling with snowball referral among parents; eligibility criteria for each cohort stated (Section 2.3).
11	Method of approach	How were participants approached?	Parents screened and referred by treating clinicians in three hospital units, then screened again by the researcher; clinicians approached through heads of department; practitioners through the gatekeepers of two organisations. All approaches were face-to-face (Sections 2.2, 2.3).
12	Sample size	How many participants were in the study?	24 parents and caregivers, 10 clinicians, 12 traditional health practitioners, 12 religious leaders; rationale by information power (Section 2.3, Table 2).
13	Non-participation	How many people refused to participate or dropped out? Reasons?	Refusals not systematically recorded; one father withdrew from a joint interview; most Hindu and Muslim representatives invited by the interfaith body did not respond (Sections 2.3, 4.4).
Setting			
14	Setting of data collection	Where was the data collected?	Parents and clinicians: research office at Chris Hani Baragwanath Academic Hospital; focus groups: venues identified by each organisation as convenient to members (Sections 2.2, 2.4).
15	Presence of non-participants	Was anyone else present besides the participants and researchers?	The research assistant was present in interviews and focus groups. No one else, for example children or organisation officials, was present.

No.	Item	Guide question or description	Reported in (revised manuscript)
16	Description of sample	What are the important characteristics of the sample?	Table 2 and accompanying text; limits of the demographic form stated (Sections 2.3, 4.4).
Data collection			
17	Interview guide	Were questions, prompts, guides provided by the authors? Was it pilot tested?	Three guides developed from the literature, reviewed by the doctoral supervisor and approved by the ethics committee; domains summarised in Table 3 and full guides in Supplementary File 3 (Section 2.4). No pilot testing was undertaken.
18	Repeat interviews	Were repeat interviews carried out?	No.
19	Audio/visual recording	Did the research use audio or visual recording?	Audio recording of interviews and focus groups with permission (Section 2.4).
20	Field notes	Were field notes made during and/or after the interview or focus group?	Reflexive journal kept throughout, recording interaction dynamics not captured on audio (Sections 2.4, 2.6).
21	Duration	What was the duration of the interviews or focus group?	Focus groups: two to three hours (Section 2.4).
22	Data saturation	Was data saturation discussed?	Saturation is not claimed; sample adequacy is argued by information power (Section 2.3).
23	Transcripts returned	Were transcripts returned to participants for comment and/or correction?	No (Sections 2.6, 4.4).
**Domain 3: Analysis and findings**			
Data analysis			
24	Number of data coders	How many data coders coded the data?	One coder (AS) in the first round; recoding by CM in the second round, checked against the transcripts by AS (Sections 2.5.1, 2.5.2).
25	Description of the coding tree	Did authors provide a description of the coding tree?	Cohort-level theme sets in the first round (Section 2.5.1; Simelane, 2021); the route-of-administration taxonomy and its categories in the second round (Section 2.5.2, Table 1).
26	Derivation of themes	Were themes identified in advance or derived from the data?	First round inductive; second round deductive framework of routes with categories added inductively where the data did not fit (Sections 2.5.1, 2.5.2).
27	Software	What software, if applicable, was used to manage the data?	None; manual coding in the first round and a spreadsheet linking entries to

No.	Item	Guide question or description	Reported in (revised manuscript)
			extracts in the second (Sections 2.5.1, 2.5.2).
28	Participant checking	Did participants provide feedback on the findings?	No (Sections 2.6, 4.4).
Reporting			
29	Quotations presented	Were participant quotations presented to illustrate the themes/findings? Was each quotation identified?	Yes; verbatim extracts identified by participant code (P1 to P24, HP1 to HP10) or by focus group (Sections 2.5, 3.2 to 3.9).
30	Data and findings consistent	Was there consistency between the data presented and the findings?	Every entry in Table 1 is linked to a source extract; Section 4.1 restates findings without inference, and interpretation is separated into Section 4.2 (Sections 2.5.2, 4.1, 4.2).
31	Clarity of major themes	Were major themes clearly presented in the findings?	Results organised by route of administration with cross-cutting findings (Sections 3.1 to 3.9, Table 1).
32	Clarity of minor themes	Is there a description of diverse cases or discussion of minor themes?	Divergent accounts reported, including the minority of parents describing change after prayer and the within-group challenge to a practitioner's knowledge (Sections 3.7, 3.8); divergence across cohorts treated as informative (Sections 2.6, 3.1).

Tong, A., Sainsbury, P., & Craig, J. (2007). Consolidated criteria for reporting qualitative research (COREQ): A 32-item checklist for interviews and focus groups. International Journal for Quality in Health Care, 19(6), 349–357. https://doi.org/10.1093/intqhc/mzm042
